# Combined gestational age and serum fucose for early prediction of risk for bronchopulmonary dysplasia in premature infants

**DOI:** 10.1186/s12887-024-04556-x

**Published:** 2024-02-12

**Authors:** Liangliang Li, Shimin Xu, Miaomiao Li, Xiangyun Yin, Hongmin Xi, Ping Yang, Lili Ma, Lijuan Zhang, Xianghong Li

**Affiliations:** 1 Division of Neonatology, The Affiliated Hospital of Qingdao University, Shandong, China; 2 Division of Neonatology, Beijing jingdu Children’s Hospital, Beijing, China; 3 Department of Medical Genetic, The Affiliated Hospital of Qingdao University, Shandong, China

**Keywords:** Bronchopulmonary dysplasia, Serum monosaccharides, Monosaccharide composite (MC), High-performance liquid chromatography, Prediction model

## Abstract

**Objective:**

As the predominant complication in preterm infants, Bronchopulmonary Dysplasia (BPD) necessitates accurate identification of infants at risk and expedited therapeutic interventions for an improved prognosis. This study evaluates the potential of Monosaccharide Composite (MC) enriched with environmental information from circulating glycans as a diagnostic biomarker for early-onset BPD, and, concurrently, appraises BPD risk in premature neonates.

**Materials and methods:**

The study incorporated 234 neonates of ≤32 weeks gestational age. Clinical data and serum samples, collected one week post-birth, were meticulously assessed. The quantification of serum-free monosaccharides and their degraded counterparts was accomplished via High-performance Liquid Chromatography (HPLC). Logistic regression analysis facilitated the construction of models for early BPD diagnosis. The diagnostic potential of various monosaccharides for BPD was determined using Receiver Operating Characteristic (ROC) curves, integrating clinical data for enhanced diagnostic precision, and evaluated by the Area Under the Curve (AUC).

**Results:**

Among the 234 neonates deemed eligible, BPD development was noted in 68 (29.06%), with 70.59% mild (48/68) and 29.41% moderate-severe (20/68) cases. Multivariate analysis delineated several significant risk factors for BPD, including gestational age, birth weight, duration of both invasive mechanical and non-invasive ventilation, Patent Ductus Arteriosus (PDA), pregnancy-induced hypertension, and concentrations of two free monosaccharides (Glc-F and Man-F) and five degraded monosaccharides (Fuc-D, GalN-D, Glc-D, and Man-D). Notably, the concentrations of Glc-D and Fuc-D in the moderate-to-severe BPD group were significantly diminished relative to the mild BPD group. A potent predictive capability for BPD development was exhibited by the conjunction of gestational age and Fuc-D, with an AUC of 0.96.

**Conclusion:**

A predictive model harnessing the power of gestational age and Fuc-D demonstrates promising efficacy in foretelling BPD development with high sensitivity (95.0%) and specificity (94.81%), potentially enabling timely intervention and improved neonatal outcomes.

## Introduction

Bronchopulmonary Dysplasia (BPD) is a chronic pulmonary disorder prevalent in very low birth weight infants (VLBWIs; 1000 g ≤ birth weight<1500 g) and extremely low birth weight infants (ELBWIs; birth weight<1000 g). This condition, instigated by impaired lung development and injurious pulmonary responses in preterm infants, imposes severe consequences such as long-term ventilatory reliance, a high mortality rate, and increased susceptibility to lower respiratory infections, airway hyper-reactivity, and growth retardation, thereby drastically undermining their quality of life. With advancements in perinatal medicine improving the survival rates of VLBWIs and ELBWIs, there is a consequent increase in BPD incidence correlating with decreasing birth age and birth weight [1, 2].

The multifaceted etiology and pathogenesis of BPD encompass various pathogenic contributors that instigate lung injury and abnormal reparative responses. The diagnosis, as per existing criteria, can only be established at the corrected age of 36 weeks, and the lack of specific treatment modalities underscores the imperative need for sensitive early biomarkers for BPD [3]. Metabolomics assays have evidenced altered glucose, lipid, and amino acid metabolism in preterm infants who develop BPD, suggesting that dysregulated glucose metabolism may serve as a predictive marker for BPD onset in preterm neonates [4, 5].

Saccharides, primarily in the form of glycans, execute biological functions as polysaccharide complexes. Lung epithelial cells boast complex carbohydrate coatings, or glycans, which bear direct or indirect relations to cell differentiation. Polysaccharide complexes, consisting chiefly of glycoproteins, proteoglycans, and glycolipids, are pervasive in cells and the extracellular matrix. A glycan represents a polysaccharide chain constituted by multiple monosaccharides linked by glycosidic bonds, with known monosaccharides including fucose (Fuc), galactose (Gal), galactosamine (GalN), glucose (Glc), glucosamine (GlcN), mannose (Man), xylose (Xyl), glucuronic acid (GlcA), iduronic acid (IdoA), N-acetylglucosamine (GlcNAc), and sialic acid (SA).

Existing reports underscore the pivotal role of glycans in lung development. Animal studies demonstrate that glycan deficiency culminates in pulmonary tissue degradation and impaired lung development [6]. Moreover, the absence of fucose inhibits the generation of secretory cells indispensable for airway development [7, 8]. The intimate relationship between glycans and lung disease pathogenesis has been well-documented [9, 10]. Glycan degradation is implicated in pulmonary injury and influences its prognosis. In acute lung injury, the cellular glycan structures can be shed into the bloodstream, hence, any aberration in blood glycan composition can serve as a marker of pathology. Therefore, variations in monosaccharide content in premature infants may offer an efficient approach for early BPD diagnosis in this vulnerable population.

In this study, we analyzed the concentrations of serum-free monosaccharides and degraded monosaccharides in premature infants through High-performance Liquid Chromatography (HPLC), looking for the novel diagnostic biomarker for BPD. Moreover, we integrating clinical data for enhanced diagnostic precision. This study aspired to construct an early diagnostic model to assess the risk and improve the prognosis of Bronchopulmonary Dysplasia (BPD).

## Materials and methods

### Subjects

The criteria for participant inclusion were as follows: (1) Gestational age ≤ 32 weeks; (2) Hospital admission within 1 hour post-birth, with a subsequent hospital stay exceeding 28 days; (3) Complete clinical data. Exclusionary factors included patients with chromosomal abnormalities, inherited metabolic diseases, or congenital developmental malformations. Our study included a cohort of 234 preterm infants. The research spanned from June 2020 to October 2023. 4 ml fasting venous blood samples from premature infants were collected when they were 1 week after birth, and centrifuged for 5 min at 13000 r/min. We took 200 μl serum and preserved it bellow at − 80 °C for the following analysis. At the same time, we collected detailed clinical information of all subjects occurred during hospitalization. In accordance with the Bronchopulmonary Dysplasia definition and grading criteria revised by the National Institute of Child Health and Human Development (NICHD) in 2018, these patients were classified into a non-BPD group (*n* = 166) and a BPD group (*n* = 68), with 48 designated as mild BPD (Grade I), and 20 as moderate to severe BPD (Grades II, III or IIIA) [3].

The study received approval from the Medical Ethics Committee of Qingdao University’s Affiliated Hospital (QYFY WZLL 28446), with informed consent obtained from all participating guardians.

### Serum free monosaccharide assessment

For each serum sample, add 5 μL of Rhamnose (Rha) (1 mg/mL), followed by 10 μL of ultrapure water, 20 μL of Sodium Hydroxide (NaOH) solution (1/88.2), and 20 μL of 0.5 mol/L 1-phenyl-3-methyl-5-pyrazolone (PMP) solution. Ensure thorough mixing. Execute PCR derivation at 70 °C for 40 minutes. Subsequently, add 20 μL of either glacial acetic acid (1/20) or 0.2 mol/L ammonium acetate solution. Carry out purification through dual rounds of chloroform extraction and centrifugation at 13,300 rpm for 15 minutes. Extract 50 μL of the supernatant solution for direct use in HPLC analysis.

### Serum monosaccharide degradation determination

For each serum sample, 5 μL was combined with Rha (1 mg/mL), 10 μL of HCl (6 mol/L), and following mixing, it underwent PCR degradation (100 °C for 10 min). Subsequent addition of (1/6.3) NaOH solution and 0.5 mol/L PMP solution occurred before carrying out PCR derivatization and purification as described in 2.2. A 50 μL aliquot of the supernatant was used for HPLC analysis. This method, known as sugar fingerprinting technology, is a patented technique of our laboratory [11].

### Monosaccharide standards and HPLC chromatograms for serum free monosaccharide and degraded monosaccharide

A standard mixed stock solution was prepared using 1 mg/mL of Mannose (Man), Glucosamine (GlcN), Rha, GlcNAc, Glucose (Glc), Galactose (Gal), and 0.125 mg/mL of Galactosamine (GalN), GlcA, Xyl, Fucose (Fuc). Following 2-fold gradient dilution, the standards, ranging from 0.5 to 0.0156 mg/mL (Man, GlcN, Rha, GlcNAc, Glc, Gal), and 0.0625 to 0.0020 mg/mL (GalN, GlcA, Xyl, Fuc), were obtained.

**Fig. 1:**
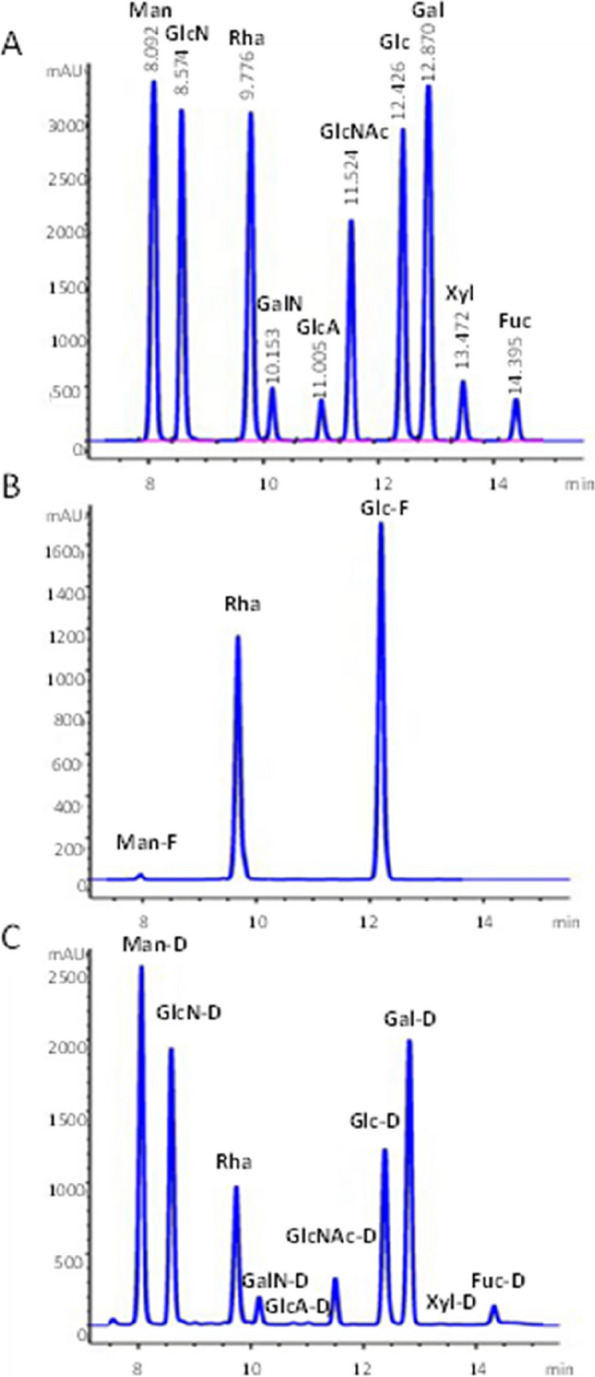
**A** HPLC chromatogram of monosaccharide standard; **B** HPLC chromatogram of serum free monosaccharides. **C** HPLC chromatogram of serum degraded monosaccharides

Rha was utilized as the internal standard to maintain the consistency of experimental operations. The monosaccharide standard mixture underwent HPLC analysis, producing the HPLC chromatogram depicted in Fig. 1A. Using the peak areas of various monosaccharide standard concentration gradients, the standard curve and regression equation were established. HPLC was used for the analysis of serum-free monosaccharides and degraded monosaccharides, obtaining the corresponding HPLC chromatograms (Fig. 1B and C). These data allowed for a precise calculation of each monosaccharide or degraded monosaccharide concentration for further analysis.

### Statistical methods

Data was processed using SPSS 25.0, and figures were generated with GraphPad Prism 8. Continuous variables adhering to the normal distribution were analyzed using the t-test and expressed as mean ± standard deviation (x ± s). Non-normal variables were subjected to the Mann-Whitney U test and presented as median and interquartile range [M (Q1, Q3)]. Qualitative data, reported as the case number and percentage, were assessed using the chi-square or Fisher’s exact test. The diagnostic potential of distinct monosaccharides for BPD was examined via ROC curves, with diagnostic efficacy evaluated using the AUC. A *p*-value less than 0.05 denotes statistical significance.

## Results

### Clinical data comparison between BPD and non-BPD groups

The study comprised 234 subjects, 166 in the non-BPD group and 68 in the BPD group, yielding a BPD incidence of 29.06%. Mild BPD accounted for 70.59% (48/68) within the BPD group, while moderate or severe BPD made up 29.41% (20/68).

Clinical data comparisons revealed lower gestational age (*P* = 0.000) and birth weight (*P* = 0.000) in the BPD group than in the non-BPD group. Both invasive and non-invasive ventilation durations were longer in the BPD group (*P* = 0.000). The BPD group exhibited a higher prevalence of Ductus Arteriosus (PDA) and gestational hypertension (*P* = 0.01). No statistically significant differences in sex, caesarean section, prelabor rupture, gestational diabetes mellitus, Small for Gestation (SGA), septicemia, Extrauterine Growth Restriction (EUGR), Pulmonary Surfactant (PS), and Respiratory Distress Syndrome (RDS) were found (*P > 0.05*) (Table 1).

**Table 1:** Clinical data comparison between BPD and Non-BPD Groups

factor	M ± SD or median (IQR) or n (%)		
	non-BPD (*n* = 166)	BPD (*n* = 68)	*P* Value
Gestational age (weeks)	31.0 (30.1, 31.55)	27.5(26.3, 28.7)	0.000
Birth weight (g)	1568.11 ± 217.37	1052.392 ± 185.61	0.000
gender			0.284
man	105 (63.3)	48 (70.6)	
women	61 (36.7)	20 (29.4)	
Cesarean	117 (70.5)	42 (62.8)	0.242
Premature rupture of membranes	62 (37.3)	30 (44.1)	0.336
Placental abnormalities	28 (16.9)	15 (22.1)	0.352
Hypertension in pregnancy	29 (17.5)	28 (41.2)	0.000
Gestational diabetes	44 (26.5)	18 (26.5)	0.996
SGA	6 (3.6)	1 (1.5)	0.382
Invasive mechanical ventilation time (days)	1.35 ± 0.55	5.70 ± 2.65	0.000
Non-invasive ventilation time (days)	9.31 (6.59, 12.38)	46.85(35.23, 60.28)	0.000
PDA	60 (36.1)	50 (73.5)	0.001
septicemia	28 (16.9)	17 (25.0)	0.152
EUGR	50 (30.1)	38 (55.9)	0.157
PS	37 (69.8)	46 (76.7)	0.439
RDS	162(97.6)	68 (100.0)	1.000

*SGA* small for gestational age, *PDA* ductus arteriosus, *EUGR* extrauterine growth restriction, *PS* pulmonary surfactant, *RDS* respiratory distress syndrome, *M ± SD* mean ± standard deviation, *IQR* interquartile range

### Analysis of the diagnostic efficacy of free monosaccharides and degraded monosaccharides in serum

We examined the concentration disparity of free and degraded monosaccharides in serum between the BPD and non-BPD groups. The BPD group exhibited significantly higher concentrations of Glc-F (*P* = 0.000), Man-F(*P* = 0.000), Man-D(*P* = 0.000), GalN-D(*P* = 0.001), Glc-D(*P* = 0.000), Gal-D(*P* = 0.015), and Fuc-D (*P* = 0.000). No statistically significant differences were observed in G/M and GlcN-D concentrations (*P > 0.05*) (Table 2).

**Table 2:** Comparison of monosaccharide content in the BPD and non-BPD groups

monosaccharide (μmol/L)	M ± SD or median (IQR)		
	non-BPD group (*n* = 166)	BPD group (*n* = 68)	*P* Value
Glc-F	5972.41 (3756.13, 9249.05)	9223.37 (8091.32, 10,355.42)	0.000
Man-F	80.38 (38.15, 115.02)	115.66 (102.97, 142.04)	0.000
G/M	85.78 (74.14, 90.14)	76.02 (66.11, 88.66)	0.216
Man-D	1168.22 (955.78, 1377. 26)	1443.96 (1015.67, 1572.73)	0.000
GlcN-D	1098.22 ± 318.32	1201.42 ± 339.22	0.172
GalN-D	606.04 (572.75, 688.42)	721.44 (611.08, 826.53)	0.001
Glc-D	5003.27 (2662.12, 5535.49)	5608 51 (3202.10, 6289.47)	0.000
Gal-D	1261.01 ± 138.04	1303.93 ± 312.34	0.015
Fuc-D	1158.27 (976.12, 1363.21)	1509.55 (1204.46, 1860.00)	0.000

*Glc-F* free glucose, *Man-F* free mannose, *G/M* free glucose/ free mannose, *Man-D* degraded mannose, *GlcN-D* degraded glucosamine, *GalN-D* degraded galactosamine, *Glc-D* degraded glucose, *Gal-D* degraded galactose, *Fuc-D* degraded fucose, *M ± SD* mean ± standard deviation, *IQR* interquartile range

**Fig. 2:**
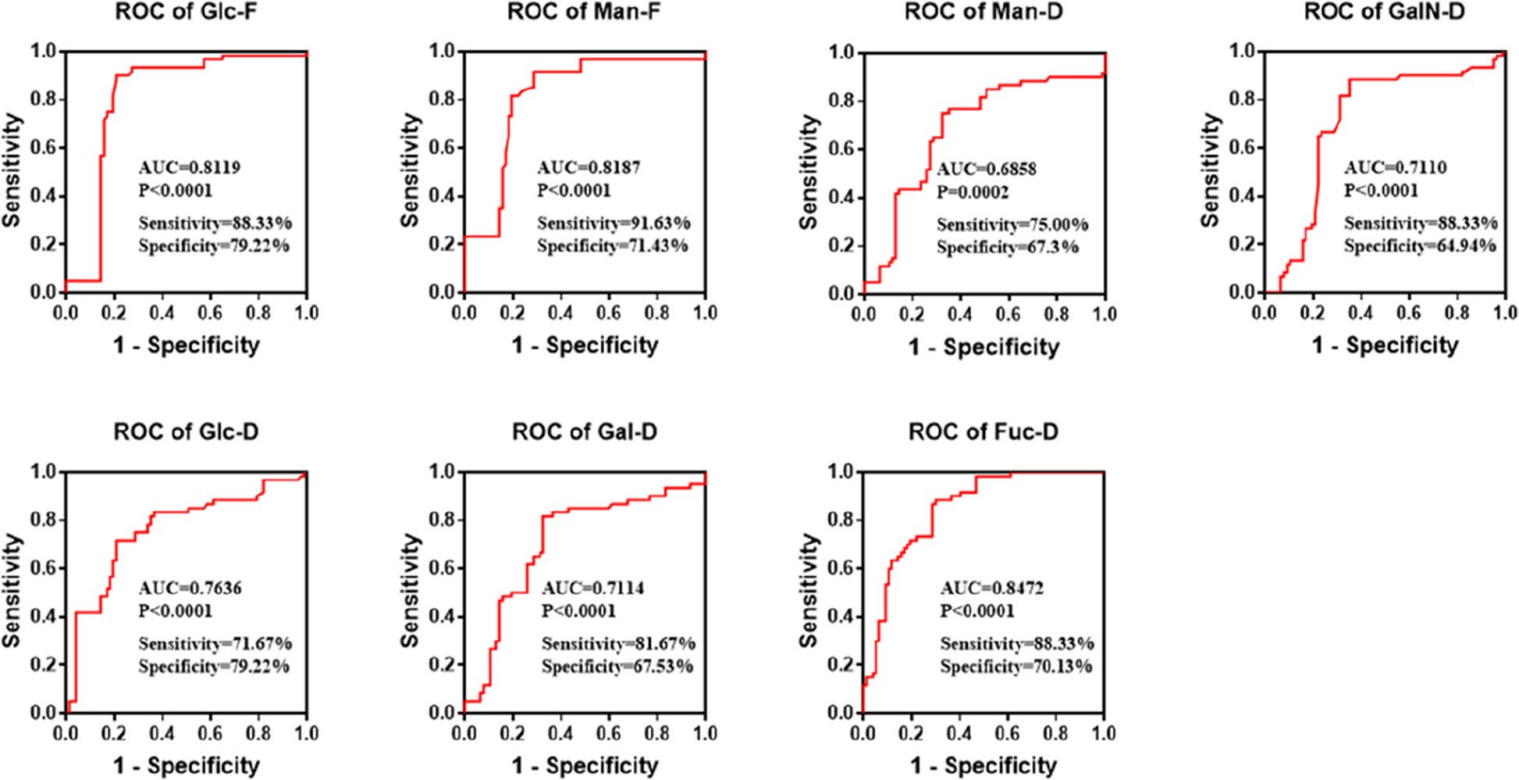
ROC curve of serum free and degraded monosaccharides

Monosaccharides displaying significant differences were subjected to ROC curve analysis, with diagnostic efficacy evaluated by AUC. The AUCs for Glc-F, Man-F, Man-D, GalN-D, Glc-D, Gal-D, and Fuc-D were 0.8119, 0.8187, 0.6858, 0.7110, 0.7636, 0.7114 and 0.8472 respectively. Notably, Glc-F (sensitivity: 88.33%, specificity: 79.22%), Man-F (sensitivity: 91.63%, specificity: 71.43%), and Fuc-D (sensitivity: 88.33%, specificity: 70.13%) demonstrated superior diagnostic performance (Fig. 2).

### Development of a BPD prediction model in premature infants

**Fig. 3:**
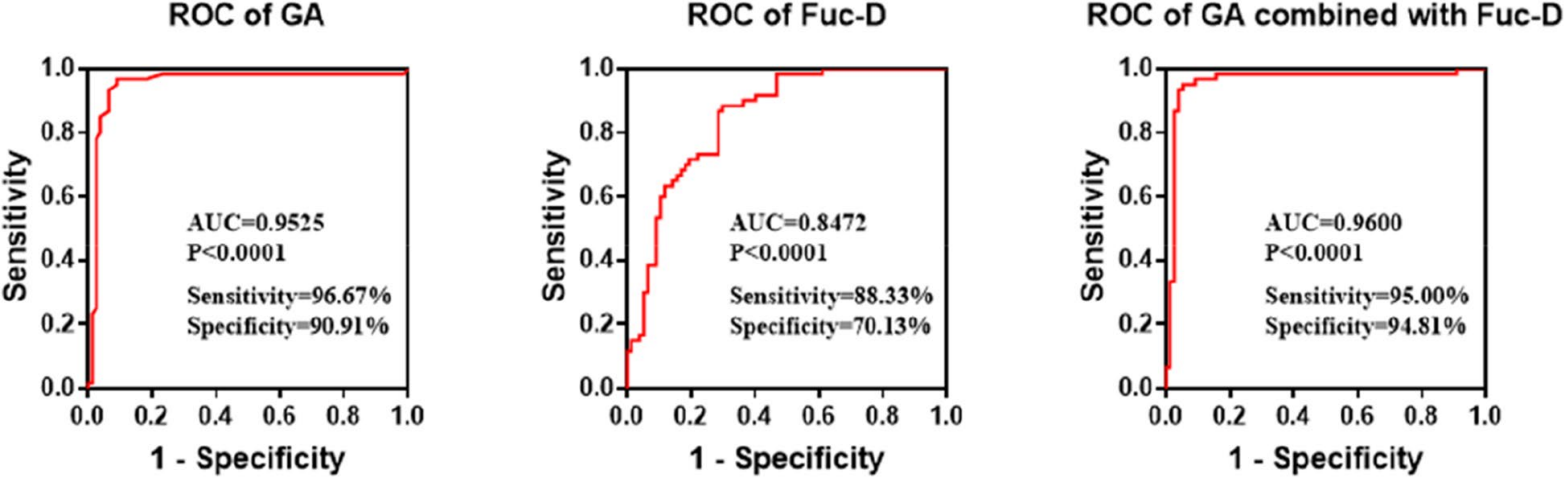
Efficacy evaluation of the BPD prediction model

Considering the disparities in clinical data and monosaccharide concentrations between the BPD and non-BPD groups, a predictive model Z = 123.975–4.325*gestational age + 0.024*Fuc-D was devised via logistic regression analysis. In comparison to individual factor predictive models, the amalgamation of gestational age and Fuc-D significantly bolstered the diagnostic efficiency, yielding an AUC of 0.96, sensitivity of 95.0%, and a specificity of 94.81%. Consequently, the predictive model, contingent on gestational age and Fuc-D offered substantial diagnostic value for BPD in premature infants (Fig. 3).

### Comparative analysis of serum free and degraded monosaccharides in mild BPD vs. moderate to severe BPD

Statistical examination revealed no significant differences in Glc-F, Man-F, G/M, Man-D, GlcN-D, GalN-D and Gal-D levels between the mild BPD group and the moderate-to-severe BPD group (*P > 0.05*). Nevertheless, the concentrations of Glc-D (*P* = 0.000) and Fuc-D (*P* = 0.005) were lower in the moderate-to-severe BPD group compared to the mild BPD group, a difference that proved statistically significant. (Table 3).

**Table 3:** Comparative analysis of serum free and degraded monosaccharides in mild BPD vs. moderate to severe BPD group

monosaccharide (μmol/L)	M ± SD or median (IQR)		
	Mild BPD group (*n* = 48)	Moderate to severe BPD group (*n* = 20)	*P* Value
Glc-F	12,517.82 (10,531.16, 12,858.16)	12,539.86 (11,375.43, 13,038.09)	0.489
Man-F	142.21 (137.95, 165.95)	125.68 (102.97, 181.78)	0.597
G/M	86.97 ± 12.32	89.29 ± 12.33	0.571
Man-D	1610.46 ± 514.94	1637.80 ± 195.99	0.767
GlcN-D	1204.70 ± 395.95	1193.78 ± 145.52	0.877
GalN-D	826.73 (721.41, 842.98)	822.48 (721.44, 845.81)	0.221
Glc-D	7198.84 (6217.53, 7593.44)	4747.59 (3152.51, 5989.24)	0.000
Gal-D	1265.76 (1139.13, 1503.94)	1296.37 (1015.24, 1426.44)	0.079
Fuc-D	1593.74 (1310.75, 1866.99)	1090.31 (1019.32, 1240.93)	0.005

*Glc-F* free glucose, *Man-F* free mannose, *G/M* free glucose/ free mannose, *Man-D* degraded mannose, *GlcN-D* degraded glucosamine, *GalN-D* degraded galactosamine, *Glc-D* degraded glucose, *Gal-D* degraded galactose, *Fuc-D* degraded fucose*, M ± S*D mean ± standard deviation, *IQR* interquartile range

## Discussion

Bronchopulmonary Dysplasia (BPD) arises due to an amalgamation of adverse influences during lung development, such as infection, volume injury, barotrauma, hyperoxia injury, and Patent Ductus Arteriosus (PDA), alongside genetic predispositions [12]. With intrinsically immature lung development, premature infants inherently bear a higher susceptibility to BPD. Our study identified a BPD frequency of 29.06%. However, the global incidence of BPD among extremely premature infants from 2006 to 2017 oscillated between 10 and 89%, a variability likely attributed to discrepancies in gestational age, birth weight, clinical diagnostic parameters, and preterm infant management strategies [13].

The comparative analysis of clinical data between BPD and non-BPD groups uncovered statistically significant differences in gestational age, birth weight, duration of invasive and non-invasive mechanical ventilation, PDA, and gestational hypertension. Predominantly, BPD occurs in very low birth weight infants (VLBWI) and extremely low birth weight infants (ELBWI) with gestational ages less than 32 weeks. The incidence rate exhibited a negative correlation with gestational age and birth weight (Table 1) [14]. Premature infants’ lungs, typically at tubular or vesicle stages of development at birth, exhibit deficient synthesis and secretion of pulmonary surfactant, diminished lung compliance, and weak antioxidant capabilities, rendering them prone to endogenous and exogenous damage [15]. BPD ensues when alveolar and microvascular development processes are impeded [16]. Mechanical ventilation, especially in relation to volume pressure injury, has been recognized as a primary contributor to lung injury, thereby an independent risk factor for BPD [17, 18]. The connection between BPD and preeclampsia, however, remains somewhat enigmatic. Previous reports have suggested that preeclampsia can result in placental dysfunction and an imbalance between pro- and anti-angiogenic factors [19], potentially impacting fetal alveolar and lung capillary development and subsequently increasing BPD risk [20, 21]. Our findings support this hypothesis, as the incidence of maternal hypertension during pregnancy was higher in the BPD group. Moreover, the incidence of PDA was greater in the BPD group. Given the hypoplastic state of the smooth muscle and the lack of intimal cushion, an immature ductus arteriosus might not close timely after birth, causing enhanced blood flow to the lungs, pulmonary edema, lung congestion, and subsequent exacerbation of pulmonary inflammation, declining lung function, and reduced gas exchange. Although the precise role of PDA in BPD onset and progression remains elusive, the risk of BPD in premature infants with PDA was found to be 6.266 times that of non-PDA counterparts, with the BPD risk escalating alongside PDA shunt volume and duration [22, 23].

Alveolar epithelial cells and capillary endothelial cells exhibit a dense glycan structure. Under physiological conditions, this glycan structure dynamically balances synthesis and degradation, offering functions such as anti-inflammation, vascular permeability maintenance, and endothelial function protection [24]. The levels of free and degraded monosaccharides in serum mirror alterations in the structure and quantity of these glycans. It has been reported that the degradation of alveolar epithelial glycans predominantly occurs in patients with acute lung injury, causing pulmonary surfactant (PS) dysfunction and correlating with the duration of mechanical ventilation [25]. Disturbances in glycan structure and the inflammatory response form two pivotal facets of acute lung injury [26, 27]. Several studies have emphasized that concentrations of glycan degradation products rise in the peripheral blood of acute lung injury patients [28]. In the event of acute lung injury, glycan degradation can promote and augment the deformation and adhesion abilities of circulating inflammatory cells, facilitating their migration to the pulmonary interstitium and alveoli [29]. Furthermore, glycan degradation prompts the production of numerous inflammatory mediators, such as oxygen free radicals, lipids, and peptides. These mediators inflict direct damage upon alveolar epithelial cells, stromal cells, and capillary basement membranes, enhancing vascular permeability [30], impeding pulmonary blood vessel development, inciting PS inactivation, and obstructing alveolarization [31]. These inflammatory mediators also display extensive biological activity, possibly inciting their re-release [32]. The continued presence of pro-inflammatory factors and chemokines triggers an unregulated ‘waterfall’ secondary inflammatory cascade [33], leading to accelerated lung glycan degradation [34], hindered repair and reconstruction function, subsequent pulmonary vascularization disorders, and abnormal repair, ultimately culminating in BPD. In this study, the BPD group demonstrated higher concentrations of Man-D, GalN-D, Glc-D, Gal-D, Fuc-D, Man-F, and Glc-F than the non-BPD group, suggesting that monosaccharide levels in premature infants could reflect lung injury and abnormal repair processes. Our research confirmed that monosaccharide content might serve as a potent early diagnostic indicator for BPD in premature infants, with the identified monosaccharides (Man-D, GalN-D, Glc-D, Gal-D, Fuc-D, Man-F, and Glc-F) demonstrating substantial predictive value for BPD.

This study has formulated a prediction model for BPD in premature infants, utilizing logistic regression analysis based on gestational age and Fuc-D content. As a glycan structure modification, Fuc contributes unique functional properties to sugar chains and plays a role in lung development and cell differentiation regulation. Deficiency of Fuc in lungs has been associated with pulmonary dysplasia [35], and it has also been implicated in the scavenging of oxygen free radicals, exhibiting antioxidant and anti-inflammatory effects. Previous studies have shown lower levels of Fuc-D in the urine metabolites of BPD group preterm infants compared to non-BPD counterparts [36]. Our investigation noted that the concentrations of Glc-D (*P* = 0.000) and Fuc-D (*P* = 0.005) were lower in the moderate-to-severe BPD group than in the mild BPD group. It was postulated that severe BPD premature infants, with heightened oxidative stress, consume more Glc and Fuc due to the presence of a large quantity of reactive oxygen radicals, leading to severe oxidative stress responses and further damage to immature lungs [37]. Nevertheless, the precise association between serum free and degraded monosaccharide levels and varying degrees of BPD warrants additional study.

In the domain of BPD prediction models, frequent predictors include birth weight, gestational age, sex, 5-minute Apgar score, respiratory distress syndrome, mechanical ventilation, antenatal steroids, maternal hypertensive disorders, surfactant, and patent ductus arteriosus [38]. Emerging markers for BPD have been reported to encompass lung ultrasound and urinary markers [39, 40]. Approximately 30% of related studies construct prediction models via univariable analysis, potentially overlooking crucial predictors. Consequently, multi-factorial prediction models are gaining wider exploration. Cai et al. identified 10 independent risk factors to construct their model, achieving an AUC value of 0.965 (sensitivity: 93.7%; specifity: 91.3%) [23]. Jassem-Bobowicz et al. established a model based on four factors, obtaining an AUC of 0.932 [3], while Shim et al. achieved high predictability (90.8%) using clinical parameters collected within 1 hour of birth [40]. In our study, the developed prediction model, based on the combination of gestational age and Fucose, delivered an AUC of 0.96, a high sensitivity of 95.0%, and a specificity of 94.81%.

In conclusion, the link between monosaccharides and BPD was investigated for the first time, suggesting Fucose as a novel marker for BPD in premature infants. Despite promising results, the main limitation of this study lies in the small sample size. Therefore, external validation through multi-center prospective studies is indispensable to further assess the generalizability of this prediction model.

## Data Availability

The datasets used and/or analyzed during the current study are not publicly available due to the institution restriction but are available from the corresponding author on reasonable request.

## Abbreviations

BPD: bronchopulmonary dysplasia
HPLC: high-performance liquid chromatography
ROC: receiver operating characteristic
AUC: area under the curve
Fuc: fucose
Gal: galactose
GalN: galactosamine
Glc: glucose
GlcN: glucosamine
Man: mannose
Xyl: xylose
GlcA: glucuronic acid
IdoA: iduronic acid
GlcNAc: N-acetylglucosamine
SA: sialic acid

## References

[1] Gilfillan M, Bhandari A, Bhandari V (2021). Diagnosis and management of bronchopulmonary dysplasia. BMJ..

[2] Lui K, Lee SK, Kusuda S, Adams M, Vento M, Reichman B, Darlow BA, Lehtonen L, Modi N, Norman M, Hakansson S, Bassler D, Rusconi F, Lodha A, Yang J, Shah PS, International Network for Evaluation of Outcomes of neonates I (2019). Trends in outcomes for neonates born very preterm and very low birth weight in 11 high-income countries. J Pediatr..

[3] Jassem-Bobowicz JM, Klasa-Mazurkiewicz D, Zawrocki A, Stefanska K, Domzalska-Popadiuk I, Kwiatkowski S, et al. Prediction model for bronchopulmonary dysplasia in preterm newborns. Children (Basel). 2021;8(10) 10.3390/children8100886. 10.3390/children8100886 PMC8534367 34682151

[4] Yue L, Lu X, Dennery PA, Yao H (2021). Metabolic dysregulation in bronchopulmonary dysplasia: implications for identification of biomarkers and therapeutic approaches. Redox Biol..

[5] Sackstein R, Stowell SR, Hoffmeister KM, Freeze HH, Varki A, Varki A, Cummings RD, Esko JD (2022). Glycans in systemic physiology. Essentials of Glycobiology.

[6] Levick JR, Michel CC (2010). Microvascular fluid exchange and the revised Starling principle. Cardiovasc Res..

[7] Lipowsky HH (2012). The endothelial glycocalyx as a barrier to leukocyte adhesion and its mediation by extracellular proteases. Ann Biomed Eng..

[8] de Fatima MM, Martins P, Goncalves CA (2019). Presence of N-acetylglucosamine residues on the surface coating of bronchioloalveolar cells during rat postnatal development: what is their purpose?. Acta Histochem..

[9] Steppan J, Hofer S, Funke B, Brenner T, Henrich M, Martin E, Weitz J, Hofmann U, Weigand MA (2011). Sepsis and major abdominal surgery lead to flaking of the endothelial glycocalix. J Surg Res..

[10] Zhang M, Zhang Y, Ma X, Liu X, Niu M, Yao R, Zhang L (2020). Using a PCR instrument to hydrolyze polysaccharides for monosaccharide composition analyses. Carbohydr Polym..

[11] Schmidt AR, Ramamoorthy C (2022). Bronchopulmonary dysplasia. Paediatr Anaesth..

[12] Siffel C, Kistler KD, Lewis JFM, Sarda SP (2021). Global incidence of bronchopulmonary dysplasia among extremely preterm infants: a systematic literature review. J Matern Fetal Neonatal Med..

[13] Jiangsu Multicenter Study Collaborative Group for Breastmilk Feeding in Neonatal Intensive Care U (2019). Clinical characteristics and risk factors of very low birth weight and extremely low birth weight infants with bronchopulmonary dysplasia: multicenter retrospective analysis. Zhonghua Er Ke Za Zhi..

[14] Baraldi E, Filippone M (2007). Chronic lung disease after premature birth. N Engl J Med..

[15] Lignelli E, Palumbo F, Myti D, Morty RE (2019). Recent advances in our understanding of the mechanisms of lung alveolarization and bronchopulmonary dysplasia. Am J Physiol Lung Cell Mol Physiol..

[16] Thebaud B, Goss KN, Laughon M, Whitsett JA, Abman SH, Steinhorn RH, Aschner JL, Davis PG, McGrath-Morrow SA, Soll RF, Jobe AH (2019). Bronchopulmonary dysplasia. Nat Rev Dis Prim..

[17] Jensen EA, Dysart K, Gantz MG, McDonald S, Bamat NA, Keszler M, Kirpalani H, Laughon MM, Poindexter BB, Duncan AF, Yoder BA, Eichenwald EC, DeMauro SB (2019). The diagnosis of bronchopulmonary dysplasia in very preterm infants. An evidence-based approach. Am J Respir Crit Care Med..

[18] Tsatsaris V, Fournier T, Winer N (2010). Pathophysiology of preeclampsia. Ann Fr Anesth Reanim..

[19] Thebaud B, Abman SH (2007). Bronchopulmonary dysplasia: where have all the vessels gone? Roles of angiogenic growth factors in chronic lung disease. Am J Respir Crit Care Med..

[20] Soliman N, Chaput K, Alshaikh B, Yusuf K (2017). Preeclampsia and the risk of bronchopulmonary dysplasia in preterm infants less than 32 Weeks' gestation. Am J Perinatol..

[21] Clyman RI, Hills NK, Liebowitz M, Johng S (2020). Relationship between duration of infant exposure to a moderate-to-large patent ductus arteriosus shunt and the risk of developing bronchopulmonary dysplasia or death before 36 weeks. Am J Perinatol..

[22] Cai H, Jiang L, Liu Y, Shen T, Yang Z, Wang S, Ma Y (2021). Development and verification of a risk prediction model for bronchopulmonary dysplasia in very low birth weight infants. Transl Pediatr..

[23] Chignalia AZ, Yetimakman F, Christiaans SC, Unal S, Bayrakci B, Wagener BM, Russell RT, Kerby JD, Pittet JF, Dull RO (2016). The Glycocalyx and trauma: a review. Shock..

[24] Rizzo AN, Haeger SM, Oshima K, Yang Y, Wallbank AM, Jin Y, et al. Alveolar epithelial glycocalyx degradation mediates surfactant dysfunction and contributes to acute respiratory distress syndrome. JCI Insight. 2022;7(2) 10.1172/jci.insight.154573. 10.1172/jci.insight.154573 PMC8855818 34874923

[25] Zhang D, Qi BY, Zhu WW, Huang X, Wang XZ (2020). Crocin alleviates lipopolysaccharide-induced acute respiratory distress syndrome by protecting against glycocalyx damage and suppressing inflammatory signaling pathways. Inflamm Res..

[26] Yamaoka-Tojo M (2020). Endothelial glycocalyx damage as a systemic inflammatory microvascular endotheliopathy in COVID-19. Biom J..

[27] Xing K, Murthy S, Liles WC, Singh JM (2012). Clinical utility of biomarkers of endothelial activation in sepsis--a systematic review. Crit Care..

[28] Lever R, Rose MJ, McKenzie EA, Page CP (2014). Heparanase induces inflammatory cell recruitment in vivo by promoting adhesion to vascular endothelium. Am J Physiol Cell Physiol..

[29] Cao RN, Tang L, Xia ZY, Xia R (2019). Endothelial glycocalyx as a potential theriapeutic target in organ injuries. Chin Med J..

[30] Mowery NT, Terzian WTH, Nelson AC (2020). Acute lung injury. Curr Probl Surg..

[31] Alvira CM, Morty RE (2017). Can we understand the pathobiology of bronchopulmonary dysplasia?. J Pediatr..

[32] Lupu F, Kinasewitz G, Dormer K (2020). The role of endothelial shear stress on haemodynamics, inflammation, coagulation and glycocalyx during sepsis. J Cell Mol Med..

[33] LaRiviere WB, Schmidt EP (2018). The pulmonary endothelial Glycocalyx in ARDS: a critical role for Heparan sulfate. Curr Top Membr..

[34] Wang X, Inoue S, Gu J, Miyoshi E, Noda K, Li W, Mizuno-Horikawa Y, Nakano M, Asahi M, Takahashi M, Uozumi N, Ihara S, Lee SH, Ikeda Y, Yamaguchi Y, Aze Y, Tomiyama Y, Fujii J, Suzuki K, Kondo A, Shapiro SD, Lopez-Otin C, Kuwaki T, Okabe M, Honke K, Taniguchi N (2005). Dysregulation of TGF-beta1 receptor activation leads to abnormal lung development and emphysema-like phenotype in core fucose-deficient mice. Proc Natl Acad Sci U S A..

[35] Huang P, Li S, Hao H, Ma F, Xiao X (2019). Differential analysis of urine metabolites in premature infants with bronchopulmonary dysplasia within 36 hours after birth. Chin J Neonatol..

[36] Sohn MH, Kang MJ, Matsuura H, Bhandari V, Chen NY, Lee CG, Elias JA (2010). The chitinase-like proteins breast regression protein-39 and YKL-40 regulate hyperoxia-induced acute lung injury. Am J Respir Crit Care Med..

[37] Peng HB, Zhan YL, Chen Y, Jin ZC, Liu F, Wang B, Yu ZB (2022). Prediction models for bronchopulmonary dysplasia in preterm infants: a systematic review. Front Pediatr..

[38] Mohamed A, Mohsen N, Diambomba Y, Lashin A, Louis D, Elsayed Y, Shah PS (2021). Lung ultrasound for prediction of bronchopulmonary dysplasia in extreme preterm neonates: a prospective diagnostic cohort study. J Pediatr..

[39] Cui X, Fu J (2022). Urinary biomarkers for the early prediction of bronchopulmonary dysplasia in preterm infants: a pilot study. Front Pediatr..

[40] Shim SY, Yun JY, Cho SJ, Kim MH, Park EA (2021). The prediction of bronchopulmonary dysplasia in very low birth weight infants through clinical indicators within 1 hour of delivery. J Korean Med Sci..

